# LNA-anti-miR-150 ameliorated kidney injury of lupus nephritis by inhibiting renal fibrosis and macrophage infiltration

**DOI:** 10.1186/s13075-019-2044-2

**Published:** 2019-12-11

**Authors:** Junjun Luan, Jingqi Fu, Chengjie Chen, Congcong Jiao, Weiwei Kong, Yixiao Zhang, Qing Chang, Yanqiu Wang, Detian Li, Gabor G. Illei, Jeffrey B. Kopp, Jingbo Pi, Hua Zhou

**Affiliations:** 1 Department of Nephrology, Shengjing Hospital of China Medical University, Shenyang, China; 2 Department of Urology, Shengjing Hospital of China Medical University, Shenyang, China; 3 Department of Clinical Epidemiology, Shengjing Hospital of China Medical University, Shenyang, China; 4 Program of Environmental Toxicology, School of Public Health, China Medical University, Shenyang, China; 5 Viela Bio, Gathesburg, MD USA; 6 National Institute of Diabetes and Digestive and Kidney Diseases, National Institutes of Health, Bethesda, USA

**Keywords:** Lupus nephritis, miR-150 inhibitor, Fibrotic genes, Macrophages, Cytokines

## Abstract

**Background:**

The prevalence of lupus nephritis (LN) remains high despite various emerging monoclonal antibodies against with targeting systemic lupus erythematosus (SLE). Renal fibrosis is the main feature of late stage LN, and novel therapeutic agents are still needed. We previously reported that microRNA (miR)-150 increases in renal biopsies of American LN patients and that miR-150 agonist promotes fibrosis in cultured kidney cells. Presently, we aim to verify whether locked nucleic acid (LNA)-anti-miR-150 can ameliorate LN in mice and to investigate its corresponding mechanisms.

**Methods:**

We first observed natural history and renal miR-150 expression in female *Fcgr2b*^*−/−*^ mice of a spontaneously developed LN model. We then verified miR-150 renal absorption and determined the dose of the suppressed miR-150 by subcutaneous injection of LNA-anti-miR-150 (2 and 4 mg/kg). Thirdly, we investigated the therapeutic effects of LNA-anti-miR-150 (2 mg/kg for 8 weeks) on LN mice and the corresponding mechanisms by studying fibrosis-related genes, cytokines, and kidney resident macrophages. Lastly, we detected the expression of renal miR-150 and the mechanism-associated factors in renal biopsies from new onset untreated LN patients.

**Results:**

*Fcgr2b*^*−/−*^ mice developed SLE indicated by positive serum autoantibodies at age 19 weeks and LN demonstrated by proteinuria at age 32 weeks. Renal miR-150 was overexpressed in LN mice compared to wild type mice. FAM-labeled LNA-anti-miR-150 was absorbed by both glomeruli and renal tubules. LNA-anti-miR-150 suppressed the elevated renal miR-150 levels in LN mice compared to the scrambled LNA without systemic toxicity. Meanwhile, serum double strand-DNA antibody, proteinuria, and kidney injury were ameliorated. Importantly, the elevated renal pro-fibrotic genes (transforming growth factor-β1, α-smooth muscle antibody, and fibronectin) and decreased anti-fibrotic gene suppressor of cytokine signal 1 were both reversed. Renal pro-inflammatory cytokines (interferon-γ, interleukin-6, and tumor necrosis factor-α) and macrophages were also decreased. In addition, the changes of renal miR-150 and associated proteins shown in LN mice were also seen in human subjects.

**Conclusions:**

LNA-anti-miR-150 may be a promising novel therapeutic agent for LN in addition to the current emerging monoclonal antibodies, and its renal protective mechanism may be mediated by anti-fibrosis and anti-inflammation as well as reduction of the infiltrated kidney resident macrophages.

## Background

Lupus nephritis (LN) is highly prevalent, and a significant portion of LN will develop into end stage renal disease (ESRD) in systemic lupus erythematosus (SLE) [1, 2]. The risk of ESRD in class IV-LN has been as high as 44% over the past 15 years despite the current available treatment regimens including steroid and immunosuppressants as well as various monoclonal antibodies [2, 3]. Renal fibrosis is a common pathological feature of ESRD that requires renal replacement therapy and is a huge economic burden worldwide [4]. Identifying novel therapeutic agents targeting key mediators of renal fibrosis is urgently needed to improve the outcome of LN.

MicroRNAs (miRs) are increasingly recognized as critical players in the pathogenesis of kidney diseases [5–8]. We previously reported that miR-150 promoted renal fibrosis in American flared LN patients by downregulating suppressor of cytokine signal 1 (SOCS1) in kidney resident cells in vitro [9]. After which, the potential role of miR-150 as a mediator of kidney diseases is further supported by the observations that higher renal miR-150 levels are associated with worse prognosis in minimal change disease [10], higher plasma miR-150 is correlated with faster renal function decline in chronic kidney disease patients [11], urinary miR-150 is increased in patients with diabetic kidney disease [12], and miR-150 transfection to young mesangial cells promotes cell aging [13]. In addition, acute kidney injury (AKI) induced by ischemia/reperfusion (I/R) is attenuated in miR-150 knockout mice [14]*.* These findings suggest that miR-150 may be a therapeutic target for kidney diseases.

Emerging studies have demonstrated that modulating pathogenic miRs can improve various kidney diseases. For example, inhibitors of miR-21, miR-192, miR-27a, miR-215, miR-34a, miR-29a, and miR-25 ameliorate diabetic nephropathy (DN) in mice [15–21]. miR-132 antagomir reduces renal fibrosis caused by unilateral ureter obstruction in mice [22]. These reports suggest modulator of miRs might open an era of utilizing nucleic acid to treat kidney diseases. However, it remains unclear whether miR-150 inhibitor can serve as a therapeutic agent for LN.

In this study, we aimed to investigate the effect of locked nucleic acid (LNA)-anti-miR-150 on kidney injury in a spontaneous LN mouse model (*Fcgr2b*^*−/−*^ mice) and clarify the corresponding mechanisms. We are the first to study the effects of LNA-anti-miR-150 in LN.

## Methods

### LN mouse model

*Fcgr2b*^*−/−*^ mice were bred in C57BL/6 mice from Jackson Laboratory (stock no. 002848) and spontaneously developed SLE and LN [23]. Total female LN (*n* = 69) and wild type (WT) mice (*n* = 69) with age 16 to 40 weeks were used in this study. The animal experimental design was conducted in four parts including natural history study of *Fcgr2b*^*−/−*^ LN mice, the renal absorption confirmation of LNA-anti-miR-150, and dose determination, as well as the effect of LNA-anti-miR-150 on kidney injury in LN mice (Additional file 2: Figure S1a-d).

### LN patients and control subjects

A human subject research protocol was approved in advance by the Institutional Review Board of the Affiliated Shengjing Hospital of China Medical University. Renal biopsies of new onset untreated LN patients (*n* = 10) were from the Department of Nephrology. The exclusion criteria for LN were patients with age < 18 or > 75, hypertension, diabetes, possible hepatitis B infection, tumors, and pregnancy. The normal control kidney tissues (*n* = 10) with age and gender matched were from the patients with kidney tumor from the Department of Urology. The control tissue is ≥ 5 cm from the tumor [24] (Additional file 2: Figure S1e).

The examination of blood and urine samples from human subjects was measured by clinical central laboratory of the hospital.

### Animal experimental details and sample collection

#### Natural history study of *Fcgr2b*^*−/−*^ mice

Peripheral blood and urine samples were collected at age week 16, 19, 32, and 40 from female *Fcgr2b*^*−/−*^ mice (*n* = 24) and WT mice (*n* = 24) to confirm positive autoantibodies for SLE and proteinuria for LN. The kidney samples were collected after perfusion with PBS to remove intrarenal blood at age 32 and 40 weeks after proteinuria was detected. Animal body weight was measured weekly (Additional file 2: Figure S1a).

#### Confirmation of LNA-miR-150 renal absorption and effective dose determination

To verify the absorption of LNA-anti-miR-150 by the kidney, female WT mice (*n* = 9) and LN mice (*n* = 9) 32 weeks old were subcutaneously (SC) injected with FAM-labeled LNA-anti-miR-150 (2 mg/kg or 4 mg/kg) (Exiqon, MA, USA) or RNase-free sterile PBS (Exiqon, MA, USA) based on 2 mg/kg of miR-192 inhibitor effective in DN mice [16]. The kidneys were harvested 6 h after PBS or FAM-labeled-LNA-anti-miR-150 or PBS injection and were embedded in OCT (Yeasen Biotech Co, Shanghai, China) and then stored at − 20 °C for immunofluorescence staining (Additional file 2: Figure S1b).

To determine the effective dose of LNA-anti-miR-150, female WT (*n* = 24) and LN mice (*n* = 24) with age 32 weeks were treated with LNA-anti-miR-150 or scrambled LNA (2 mg/kg or 4 mg/kg SC). The kidneys were collected 6 h after PBS perfusion for verifying the suppression of renal miR-150 levels (Additional file 2: Figure S1c).

#### The effect of LNA-anti-miR-150 on kidney injury in LN mice

To investigate the effect of LNA-anti-miR-150 on kidney injury in LN mice, female WT (*n* = 12) and LN mice (*n* = 12) with age 32 weeks were treated with LNA-anti-miR-150 or scrambled LNA (2 mg/kg twice weekly, SC) for 8 weeks (Additional file 2: Figure S1d and the sequence of LNA seen in Additional file 1: Table S5). Urine and serum samples were collected and stored at − 80 °C for future measurement. PBS-perfused kidney samples were prepared as (1) formalin-fixed paraffin embedded blocks for histology examination under light microscopy, (2) OCT-fixed frozen tissue blocks and stored at − 20 °C for immunofluorescence staining, and (3) fresh frozen kidney tissue and stored at − 80 °C for future isolation of protein and RNA.

### Examination of serological and urinary chemistry

Serum anti-nuclear antibody (ANA) was measured with anti-mouse ANA IgG enzyme-linked immunosorbent assay (ELISA) kit (Alpha Diagnostic International, San Antonio, USA). Serum anti-double strand DNA (ds-DNA) was examined with an anti-mouse ds-DNA IgG ELISA kit (Cusabio, Wuhan, China) according to the manufacturer’s instructions.

A serum biochemistry panel including serum creatinine (Scr), blood urea nitrogen (BUN), alanine aminotransferase (ALT), and aspartate amino transferase (AST) was analyzed by an Architect c16000 device (Abbott, Chicago, USA).

Urinary albumin and creatinine were measured by ELISA companion kit (Exocell Inc., Philadelphia, USA) according to the manufacturer’s instructions. The urinary albumin/creatinine ratio (ACR) was expressed as microgram/milligram.

### Histology and immunostaining

Paraffin-embedded kidneys were cut at 3 μm for mouse and at 2 μm thickness for human, deparaffinized, rehydrated, and then stained with periodic acid-Schiff (PAS) or Masson-trichrome reagent for histological examination.

For immunofluorescence staining, mouse kidney slides with same pretreatment were incubated with antibodies of Complement1q (C1q), transforming growth factor-β1 (TGF-β1), α-smooth muscle antibody (α-SMA), and fibronectin (FN), SOCS1, CD19, CD3, CD4, CD8, and CD68 at 4 °C overnight followed by incubation with Alexa-568/Alexa-488 donkey anti-rabbit/anti-mouse IgG (Thermo Fisher Scientific, Rockford, USA) at room temperature (RT) for 1 h. After three washes with PBS, the slides were mounted with DAPI for 10 min. Images were captured by immunofluorescent microscopy (Nikon Corporation, Tokyo, Japan). The parameters were quantified by Image-Pro Plus 6.0 (Media cybernetics, Maryland, USA).

For immunohistochemistry (IHC) staining, human kidney sections were deparaffinized, rehydrated, and incubated in citrate buffer for 20 min at 95 °C to retrieve antigen. Nonspecific binding was blocked with 10% normal goat serum for 30 min at RT. The slides were incubated with antibodies of TGF-β1, α-SMA, FN, SOCS1, F4/80, and CD68 overnight at 4 °C, followed by incubation with biotin-conjugated goat anti-mouse/rabbit immunoglobulin IgG for 30 min at RT, and then reacted with streptavidin-conjugated peroxidase for 30 min at RT. The reaction products were visualized using a DAB kit. The six parameters on IHC were quantified by NIH Image J software as previously reported [25].

On PAS and Masson staining, we semi-quantified the glomerular and tubulointerstitial injury separately as previously described [26]. The average number of infiltrated macrophages in tubulointerstitium and glomeruli was calculated by counting 20 high power fields through CD68 immunostaining as previously reported [27].

### Immunoblotting

Frozen kidney tissues were homogenized in RIPA buffer with protease inhibitor cocktail. Protein concentrations were determined by BCA assay. Equal amount of individual protein was separated by SDS-PAGE, and the gels were transferred onto PVDF membranes (Immobilon-P, Millipore, German). After blocking with 5% milk, membranes were incubated with primary antibody against TGF-β1, α-SMA, FN, SOCS1, and F4/80, respectively, overnight at 4 °C. The blots were incubated with peroxidase-conjugated goat anti-rabbit/mouse IgG for 1 h at RT. The antibody-antigen reactions were detected by High-sig ECL Western Blotting Substrate and visualized by the Tanon 5500 imaging system. The loading variation of individual protein was normalized by α-TUBULIN or GAPDH based on the molecular weight of target proteins. The density of blots was analyzed by NIH Image J software. The protein level is expressed as the ratio of blot density of individual protein to its housekeeper (the antibodies for immunostaining and immunoblotting in Additional file 1: Table S3).

### Quantitative real-time PCR

Total RNAs were extracted from frozen mouse and human kidneys with Trizol reagent (Life Technologies, Carlsbad, USA), and the concentration was measured with Nanodrop 2000. Mouse RNA was subjected to reverse transcription using Prime Script RT Reagent Kit and followed by PCR with SYBR Premix Ex Taq (Takara, Dalian, China) for mRNA of *Tgfβ1*, *αSma*, *Fn*, *Socs1*, interferon-γ (*Infγ*), interleukin-6 (*Il6*), and tumor necrosis factor-α (*Tnfα*). Total RNAs were subjected to a similar process for miR-150. RNA was subjected to reverse transcription using TransScripit miRNA First-Strand cDNA Synthesis SuperMix (TransGen Biotech, Beijing, China) and followed by PCR with SYBR Premix Ex Taq (Takara, Dalian, China). Primers were designed using Primer Express (Applied Biosystems, Carlsbad, USA) and synthesized by Life Technologies (Shanghai, China). Real-time fluorescence was detected with QuantStudio 6 Flex quantitative PCR system (Applied Biosystems, Carlsbad, USA). *Actin*, *Sno202*, and U6 were used as endogenous controls of mRNA, mouse miR-150, and human miR-150, respectively (Additional file 1: Table S4). The levels of mRNA and miR-150 were expressed as 2^−ΔΔCt^ (ΔCt: Ct value of endogenous control gene − Ct of individual target gene).

### In situ hybridization of miR-150 in human kidney tissues

In situ hybridization of miR-150 was performed in control kidneys and biopsies of new onset LN patients by following the procedure as we had described previously [9]. Human kidney sections were deparaffinized, washed by DEPC-distilled water, permeabilized in 0.1 N HCl for 15 min at RT, and then washed in PBS followed by acetylation for 15 min and washing in SSC. The sections were covered with miR-150 probe (U6 as positive control and scrambled siRNA as negative control) and denatured at 75 °C for 12 min followed by incubation at 55 °C for 22 h. A series of post-hybridization wash in gradient SSC (2×, 1×, 0.5× and 0.25×) for 10 min each at 55 °C were conducted. Sections were placed in blocking solution for 1 h at RT followed by incubation in digoxigenin-AP Fab Fragment (1:100) for 2 h and followed by wash in PBS Tween for 15 min at RT and then wash in alkaline phosphatase buffer for 10 min at RT. Then, sections were developed with 5-bromo-4-chloro-3′-indolyphosphate/nitro blue tetrazolium chloride and counterstained with nuclear fast red.

### Statistical analysis

Statistical software SPSS 22.0 (SPSS, Chicago, USA) and Graphpad Prism 8 (Graphpad, San Diego, USA) were used for statistical analysis and graphing. Quantitative data were expressed as mean ± SD. Differences between groups were analyzed for statistical significance by two-way ANOVA or *t* test. A *p* value < 0.05 was accepted as statistically significant.

## Results

### The natural history of *Fcgr2b*^*−/−*^ LN mice

**Fig. 1:**
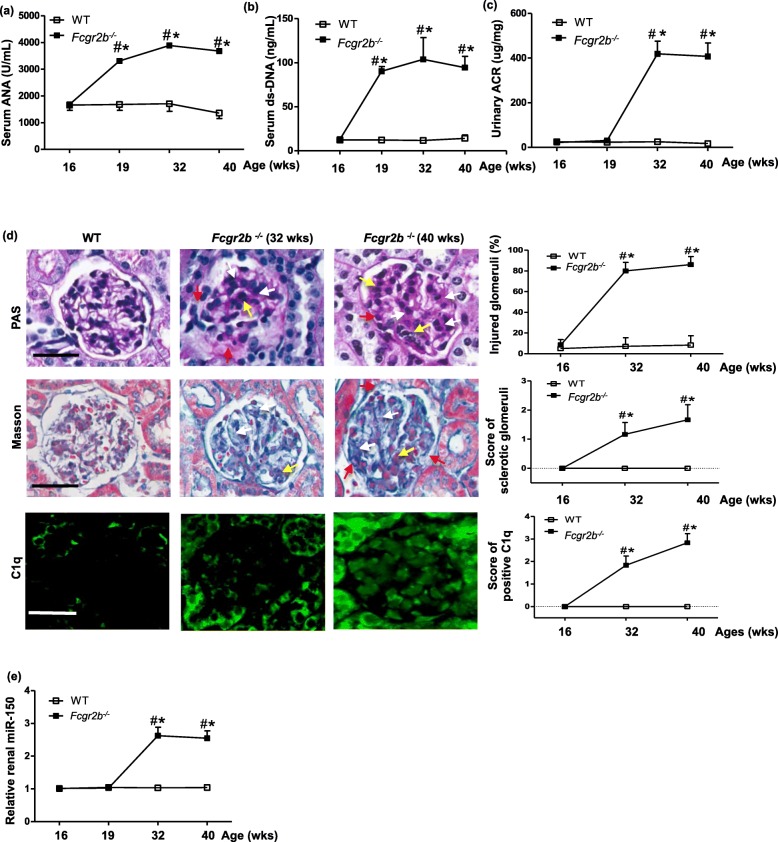
The natural history of *Fcgr2b*^*−/−*^ spontaneous LN mice. **a**, **b** The temporal levels of serum anti-nuclear antibody (ANA) and anti-double strand DNA (ds-DNA). **c** Urinary albumin/creatinine ratio (ACR) determined by ELISA in *Fcgr2b*^*−/−*^ mice from age 16 weeks to 40 weeks. **d** PAS and Masson staining of paraffin-embedded kidney sections taken from wild type (WT) and *Fcgr2b*^*−/−*^ mice at age 32 weeks and 40 weeks. Representative lobular proliferation including the increase of mesangial cells and the expansion of matrix (white arrow), endocapillary proliferation (yellow arrow), adhesion of capillary tufts with Bowman’s capsule (red arrow), and some detachment and vacuolization of podocytes. Immunofluoscent staining of C1q in frozen kidney tissue of wild type (WT) and *Fcgr2b*^*−/−*^ mice at age 32 weeks (moderately positive) and 40 weeks (fully positive). The semi-quantification of kidney injury was determined by respective scoring system. **e** The temporal levels of renal miR-150 assayed by qPCR in *Fcgr2b*^*−/−*^ mice and WT mice. Data are expressed as mean ± SD from six mice per group. Statistical significance was determined using two-way ANOVA (^#^*p* < 0.05, *Fcgr2b*^*−/−*^ vs. WT; *p < 0.05 each age of *Fcgr2b*^*−/−*^ vs. age 16 week)

Female *Fcgr2b*^*−/−*^ mice spontaneously developed into SLE at age week 19 as indicated by positive serological ANA (Fig. 1a) and ds-DNA (Fig. 1b), after which mice progressed into LN at age week 32 verified by proteinuria (Fig. 1c). The typical kidney histology features of LN mice at week 32 included glomerular lobular endocapillary proliferation, mesangial cells proliferation, mesangial matrix expansion, and adhesion of capillary tufts with Bowman’s capsules on PAS and Masson staining as well as LN-specific positive C1q on immunofluorescence staining. These morphological changes became more severe at age week 40 with demonstration of the above histology semi-quantification (Fig. 1d). The levels of renal miR-150 in LN mice significantly increased at week 32 when proteinuria was detected and remained elevated until the week 40 experimental end point (Fig. 1e).

### The renal absorption of LNA-anti-miR-150 and suppression of renal miR-150 levels

**Fig. 2:**
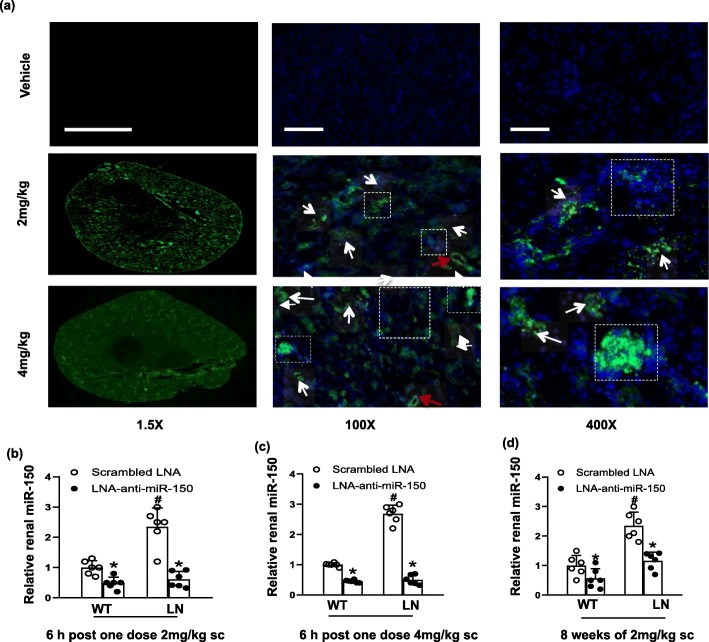
Renal absorption of LNA-anti-miR-150 and the suppression of renal miR-150. **a** Renal absorption of LNA-anti-miR-150 was detected by fluorescence of FAM-labeled LNA-anti-miR-150 at 6 h after the subcutaneous injection of 2.0 mg/kg and 4.0 mg/kg to six mice per group. Open squares represent glomeruli, white arrows represent tubules, and red arrows represent vessels (magnification × 1.5, bar = 2000 μm; magnification × 100, bar = 25 μm; magnification × 400, bar = 100 μm). **b**, **c** The levels of renal miR-150 were measured by qPCR 6 h after one dose of 2.0 mg/kg or 4 mg/kg of LNA-anti-miR-150 subcutaneous injection and **d** 8 weeks after LNA-anti-miR-150 (2 mg/kg, twice weekly for total 16 doses) administration. Data are expressed as mean ± SD from six mice per group. Statistical significance was determined using two-way ANOVA (^#^*p* < 0.05, LN vs. WT; *p < 0.05, LNA-anti-miR-150 vs. the scrambled LNA)

Since LNA-anti-miRs are potent and stable miR inhibitors [16, 28], we used LNA-anti-miR-150 to inhibit endogenous miR-150 in the present study. First, we investigated whether LNA-anti-miR-150 could be absorbed by kidney cells of mice subcutaneously injected with FAM-labeled LNA-anti-miR-150. We found that FAM-labeled LNA-anti-miR-150 was absorbed by both glomeruli and renal tubules (Fig. 2a). In addition, the renal absorption showed a dose-dependent manner 6 h after the injection of FAM-labeled LNA-anti-miR-150 at 2 mg/kg and 4 mg/kg. Since LNA-anti-miR-150 showed sufficient inhibition of renal miR-150 level in both WT and LN mice at both doses (Fig. 2b, c), we chose 2 mg/kg of LNA-anti-miR-150 (twice weekly for 8 weeks) to investigate the therapeutic effect on kidney injury. We found that renal miR-150 was inhibited in both WT and LN mice (Fig. 2d). In addition, no effect was found in the kidney, liver function, and body weight (Additional file 2: Figure S2a-e).

### The effect of LNA-anti-miR-150 on LN

**Fig. 3:**
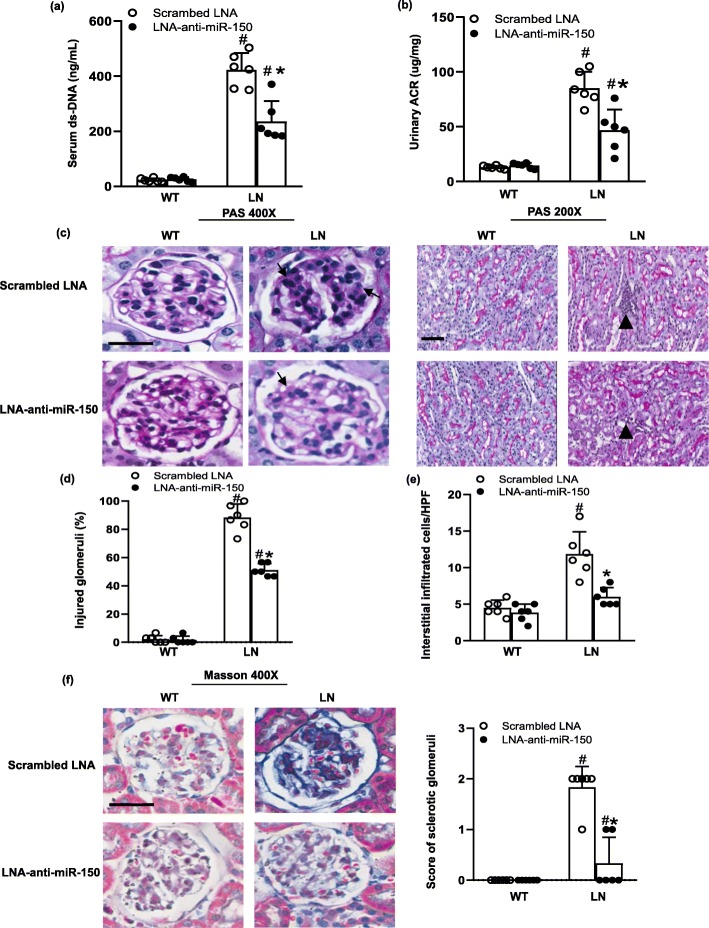
The effect of LNA-anti-miR-150 on kidney injury in LN mice. **a**, **b** Serum ds-DNA titer and urinary albumin/creatinine ratio (ACR) determined by ELISA in WT and LN mice treated with the scrambled LNA or LNA-anti-miR-150 (subcutaneous injection twice weekly for 8 weeks). **c** PAS staining of paraffin-embedded kidney sections taken from same mice showed glomerular matrix proliferation (arrow, magnification × 400, bar = 200 μm) and the tubulointerstitial infiltration of inflammatory cells (arrowhead, magnification × 200, bar = 200 μm). **d** Glomerular damages were semi-quantified as a percentage of the injured glomeruli, and **e** tubulointerstitial infiltrated inflammatory cells were counted per high power field (HPF) on PAS staining. **f** Masson staining of paraffin-embedded kidney sections taken from experimental mice and the semi-quantification score of sclerotic glomeruli (blue area, magnification × 400, bar = 200 μm). Data are expressed as mean ± SD from six mice per group. Statistical significance was determined using two-way ANOVA (^#^*p* < 0.05, LN vs. WT; *p < 0.05, LNA-anti-miR-150 vs. the scrambled LNA)

After confirming the inhibiting effect of LNA-anti-miR-150 on renal miR-150 and systemic safety, we investigated the therapeutic effect of LNA-anti-miR-150 on autoantibodies for SLE and kidney injury for LN. We found that LNA-anti-miR-150 decreased the serological ds-DNA titer (Fig. 3a) and proteinuria excretion (Fig. 3b) compared to the scrambled LNA in LN mice. Endocapillary proliferation, mesangial cell proliferation, matrix expansion, and glomerular tuft lobularization were significantly improved as shown through PAS staining (Fig. 3c) and semi-quantified percentage of injured glomeruli (Fig. 3d). Similarly, the infiltration of inflammatory cells was decreased as demonstrated by PAS staining (Fig. 3c) and semi-quantification (Fig 3e). In addition, we further evaluated the sclerotic glomeruli based on the blue area in each glomerulus on Masson staining and found that sclerotic glomeruli were also decreased with treatment of LNA-anti-miR-150 compared to the scrambled LNA in LN mice; similarly, semi-quantification score of sclerotic glomeruli showed a reduction of glomerulosclerosis by LNA-anti-miR-150 (Fig. 3f).

### The effect of LNA-anti-miR-150 on renal pro-fibrotic genes

**Fig. 4:**
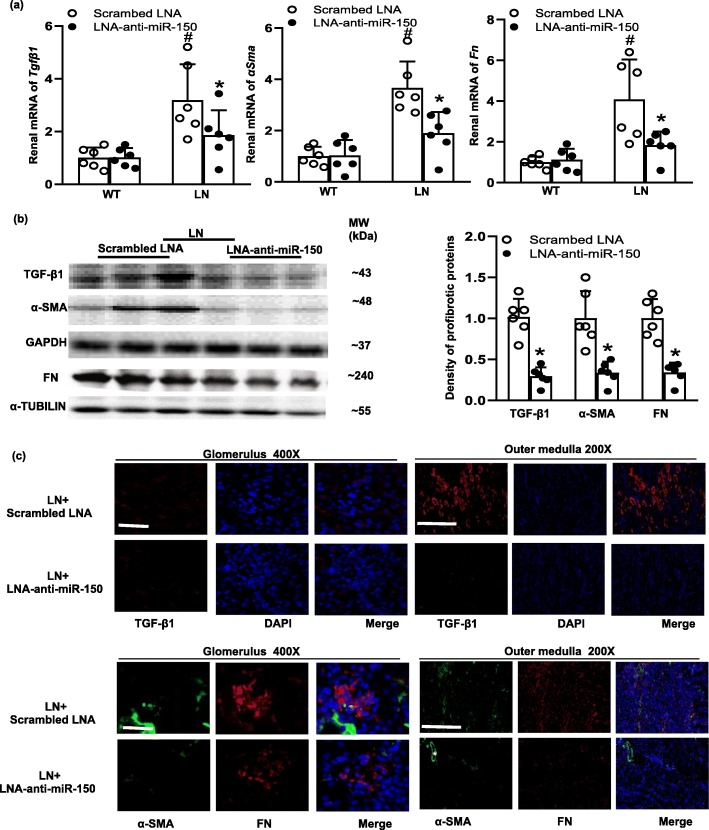
The effect of LNA-anti-miR-150 on renal pro-fibrotic genes and proteins in LN mice. **a** Renal mRNA levels of *Tgfβ1*, *αSma*, and *Fn* determined by qPCR; **b** renal protein levels of TGF-β1, α-SMA, and FN examined by western blotting and the density of the blots; and **c** immunoflurescent staining of TGF-β1 (red), α-SMA (green), and FN (red) in glomeruli (magnification × 400, bar = 30 μm) and the outer medulla (magnification × 200, bar = 100 μm) of paraffin-embedded kidney sections taken from WT and LN mice treated with the scrambled LNA or LNA-anti-miR-150 (subcutaneous injection twice weekly for 8 weeks). White asterisks represent vessels as positive control of α-SMA. Data are expressed as mean ± SD from six mice per group. Statistical significance was determined using two-way ANOVA (^#^*p* < 0.05, LN vs. WT; *p < 0.05, LNA-anti-miR-150 vs. the scrambled LNA)

Since our previous study found that renal miR-150 levels positively correlated with pro-fibrotic genes [9], we examined the effects of LNA-anti-miR-150 on renal pro-fibrotic genes. We found that mRNA levels of *Tgfβ1*, *αSma*, and *Fn* were increased in LN mice compared to WT mice. LNA-anti-miR-150 reduced the increase of these pro-fibrotic genes compared to the scrambled LNA (Fig. 4a). The same ameliorative effect of LNA-anti-miR-150 was also seen in protein levels of these pro-fibrotic genes as analyzed by western blotting (Fig. 4b) and immunostaining (Fig. 4c).

### The effect of LNA-anti-miR-150 on renal SOCS1

**Fig. 5:**
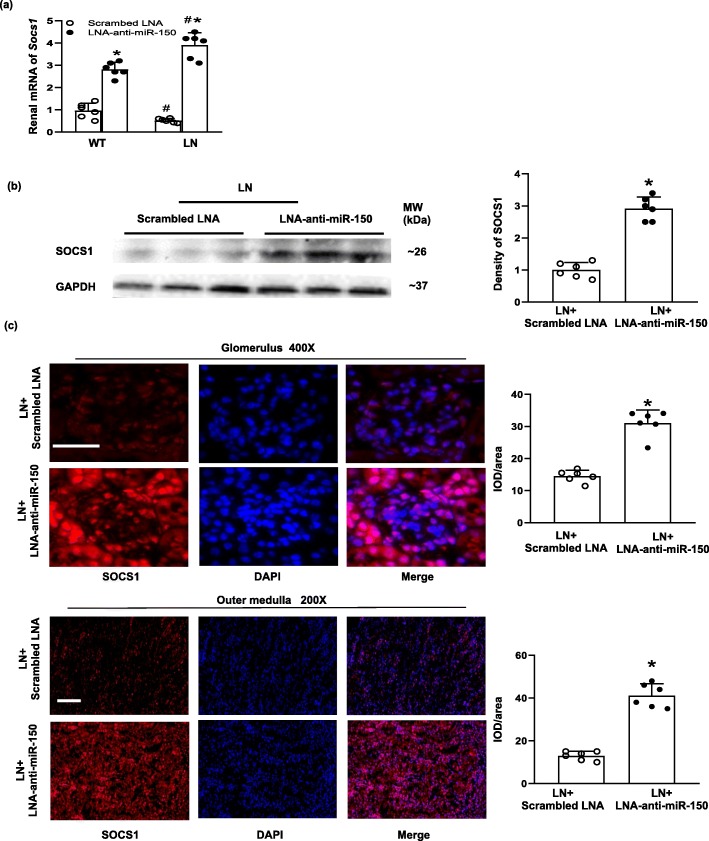
The effect of LNA-anti-miR-150 on renal SOCS1 in LN mice. **a** Renal mRNA levels of *Socs1* determined by qPCR, **b** renal protein levels of Socs1 examined by western blotting and the density of the blots, and **c** immunoflurescent staining of Socs1 and its integrated optical density per area (IOD/area) in glomeruli (magnification × 400, bar = 30 μm) and the outer medulla (magnification × 200, bar = 100 μm) of paraffin-embedded kidney sections taken from WT and LN mice treated with the scrambled LNA or LNA-anti-miR-150 (subcutaneous injection twice weekly for 8 weeks). Data are expressed as mean ± SD from six mice per group. Statistical significance was determined using two-way ANOVA in **a** and *t* test in **b**, **c** (^#^*p* < 0.05, LN vs. WT; *p < 0.05, LNA-anti-miR-150 vs. the scrambled LNA)

Anti-fibrotic SOCS1 was revealed as a direct target gene of miR-150 via analyzing luciferase activity in vitro in our previous study [9]. In the present study, we further examined the effect of LNA-anti-miR-150 on renal SOCS1 expression. We found that SOCS1 mRNA level was decreased in LN mice compared to WT mice. LNA-anti-miR-150 increased SOCS1 mRNA levels up to sixfolds compared to the scrambled LNA in both WT and LN mice (Fig. 5a). SOCS1 protein levels were increased threefold by LNA-anti-miR-150 compared to the scrambled LNA in LN mice as shown by western blotting analysis and semi-quantification of the blots (Fig. 5b). Immunostaining of SOCS1 showed the same increased changes by qPCR and western blotting (Fig. 5c).

### The effect of LNA-anti-miR-150 on renal cytokines and immune cells

**Fig. 6:**
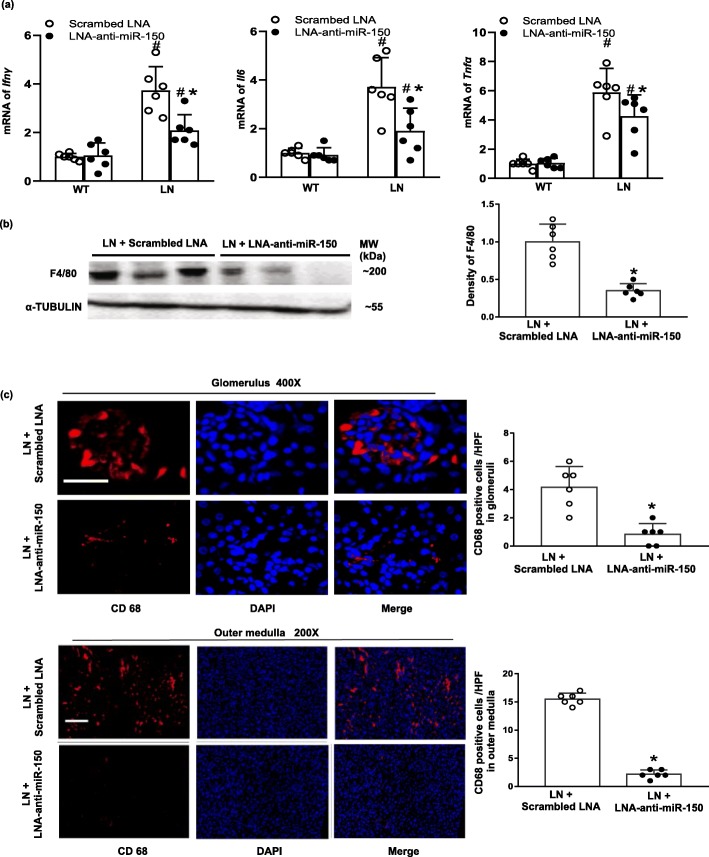
The effect of LNA-anti-miR-150 on renal pro-inflammatory cytokines and macrophages in LN mice. **a** The renal mRNA levels of cytokines including *Ifnγ*, *Il6*, and *Tnf* determined by qPCR; **b** renal protein levels of F4/80, a macrophage biomarker, examined by western blotting and the density of the blots; and **c** immunoflurescent staining of CD68, an alternative macrophage biomarker, and its integrated optical density per area (IOD/area) in glomeruli (magnification × 400, bar = 30 μm) and the outer medulla (magnification × 200, bar = 100 μm) of paraffin-embedded kidney sections taken from WT and LN mice treated with the scrambled LNA or LNA-anti-miR-150 (subcutaneous injection twice weekly for 8 weeks). Data are expressed as mean ± SD from six mice per group. Statistical significance was determined using two-way ANOVA in **a** and *t* test in **b**, **c** (^#^*p* < 0.05, LN vs. WT; *p < 0.05, LNA-anti-miR-150 vs. the scrambled LNA)

Recently, inflammation and kidney resident macrophages have become increasingly emphasized in LN pathogenesis [29, 30]. We examined renal cytokines such as *Infγ*, *Il6*, and *Tnfα* as well as kidney resident macrophages. The mRNA of these cytokines increased in LN mice compared to WT mice, and LNA-anti-miR-150 inhibited the elevation of these renal cytokines compared to the scrambled LNA (Fig. 6a). To explore the mechanisms of cytokine changes, we examined the expression of kidney resident macrophage biomarkers F4/80 and CD68. We found that LNA-anti-miR-150 decreased renal F4/80 expression in LN mice by western blotting analysis (Fig. 6b). LNA-anti-miR-150 also decreased the renal infiltration of CD68 as shown by immunostaining and semi-quantification of CD68-positive cells in glomeruli and outer medulla (Fig. 6c). B cells are a well-known source of autoantibodies in LN. However, B cells were not detected in the kidneys of LN mice (Additional file 2: Figure S3a). Similar to B cells, T cells appeared rarely and showed no difference in the kidneys of LN mice treated with LNA-anti-miR-150 or scrambled LNA as compared to the positive control spleen tissue from the same mice (Additional file 2: Figure S3b-d).

### The renal expression of miR-150 and its regulated proteins in human subjects

**Fig. 7:**
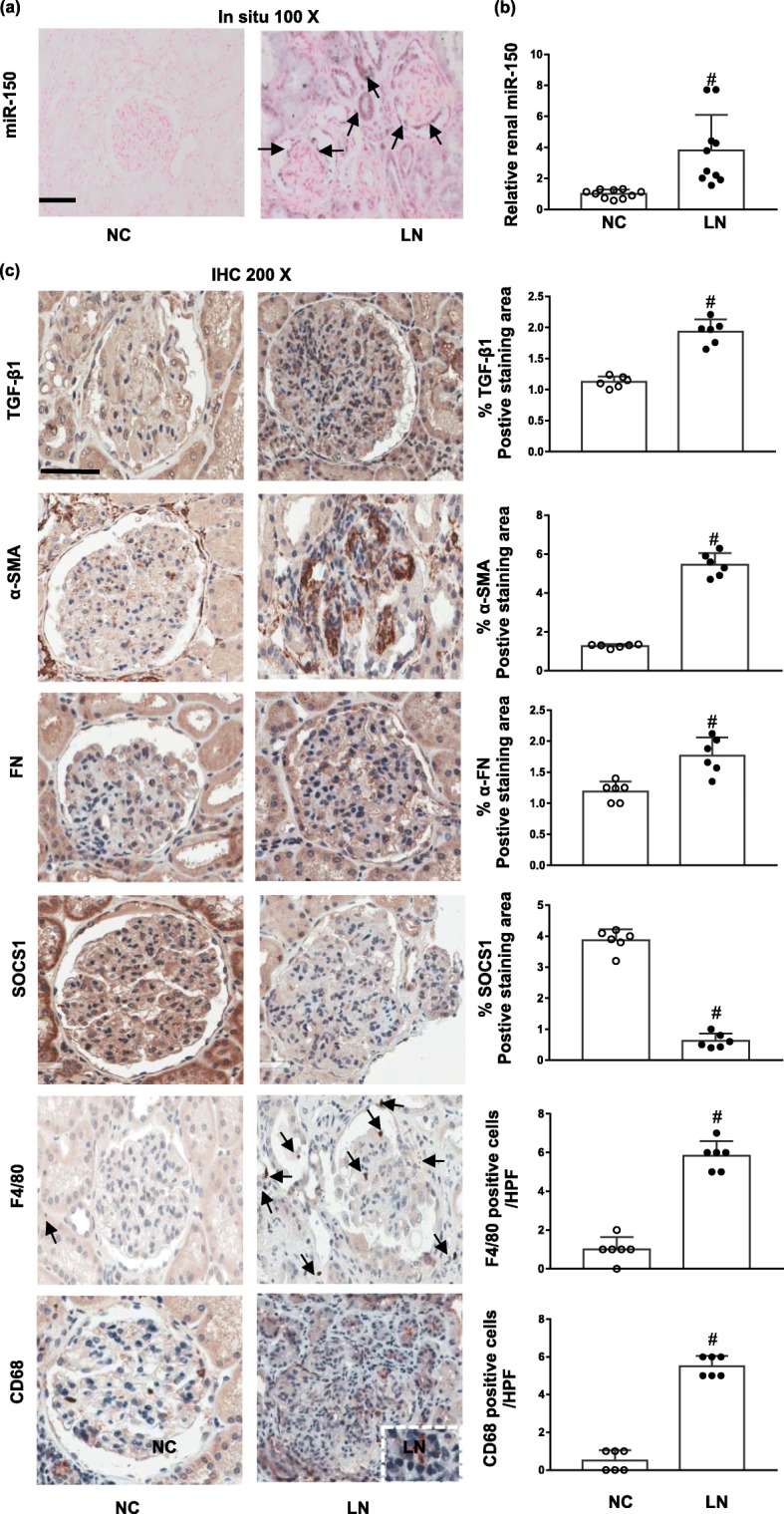
miR-150 expression in the renal biopsies from LN patients. In kidney biopsies from LN patients and normal control kidneys (NC), **a** the renal distribution of miR-150 stained by in situ hybridization (arrows, magnification × 100, bar = 100 μm) and **b** renal expression levels of miR-150 determined by qPCR; **c** the immunohistochemistry staining of fibrotic-related proteins including pro-fibrotic proteins (TGF-β1, α-SMA, and FN), anti-fibrotic protein SOCS1, and the infiltration of macrophages indicated F4/80 (arrow) and CD68 (positive tubulointerstitial CD68 in insert panel) as well as the percentage of positive staining of each above protein. Data are expressed as mean ± SD from ten subjects per group. Statistical significance was determined using *t* test (^#^*p* < 0.05, LN vs. NC, magnification × 200, bar = 50 μm)

After investigation of LNA-miR-150 in LN mice, we explored the renal expression and localization of miR-150 in human subjects including untreated new onset Chinese LN patients (Additional file 1: Table S1 and Additional file 2: Figure S4) and control patients with kidney nephrectomy (Additional file 1: Table S2). Similar to our previous results in American Caucasian LN patients [9], we found that in situ hybridization showed miR-150 mainly increased in tubular cells and moderately in podocytes (Fig. 7a). The level of miR-150 increased in renal biopsies of Chinese LN patients compared to normal control kidneys by qPCR analysis (Fig. 7b).

We further examined the renal expression of proteins regulated by miR-150 through immunochemistry staining in human kidney tissues. Similar to our findings in LN mice, we found increased renal expression TGF-β1, FN, and α-SMA and decreased SOCS1 in renal biopsies of Chinese LN patients compared to normal control kidneys. In addition, we found that F4/80 and CD68, two macrophage biomarkers, were significantly increased in the kidneys of LN patients compared to the normal control kidneys (Fig. 7c).

## Discussion

The major findings of this study include the following: (1) miR-150 increased in the kidneys of *Fcgr2b*^*−/−*^ mice, a spontaneously developed LN mouse model, and renal biopsies of new onset untreated LN patients. (2) LNA-anti-miR-150 attenuated the proteinuria and kidney injury severity compared to the scrambled LNA in LN mice. (3) LNA-anti-miR-150 decreased renal pro-fibrotic proteins and increased anti-fibrotic protein SOCS1. (4) LNA-anti-miR-150 decreased renal pro-inflammatory cytokines and the infiltration of kidney resident macrophages.

We previously found that miR-150 increased mainly in renal tubular cells and moderately in podocytes in the repeated renal biopsies of flared American LN patients and that miR-150 promoted renal fibrosis by downregulating SOCS1 through in vitro study [9]. After our report, other groups found that the deletion of miR-150 alleviates renal tubulointerstitial fibrosis in mice 8 weeks after I/R by increasing collagen type I and connective tissue growth factor [14]. Besides kidney diseases, blood miR-150 level increased in SLE patients compared to those with primary Sjogren’s syndrome [31]. These data suggest that miR-150 might be a novel therapeutic target in both SLE and LN. In this study, we aim to verify whether miR-150 inhibitor can serve as a new agent to treat LN. To do so, we selected the spontaneous SLE (Fig. 1a, b) and LN mouse model (Fig. 1c, d) that we had previously used [23] to investigate the effect of LNA-anti-miR-150, a stable type of miR inhibitor, on kidney injury, renal inflammation, and fibrosis in vivo. To our knowledge, this is the first study to explore the renal protective role of LNA-anti-miR-150 and its corresponding mechanisms. We first evaluated the safety of LNA-anti-miR-150 as a therapeutic agent. In the whole study, no damages in liver and kidney function or loss of animal body weight was shown after the treatment of LNA-anti-miR-150 in LN (Additional file 2: Figure S2a-e).

We found that renal miR-150 increased in female *Fcgr2b*^*−/−*^ LN mice at age 32 weeks (Fig. 1e). This was consistent with the elevated miR-150 in the repeated renal biopsies from American LN patients in our previous study [9]. These data provided us direct evidence to explore the effects of miR-150 inhibition in LN mice in this preclinical study. Since LNA-anti-miRs are stable and easily absorbed by kidney tissue [16], we chose LNA-anti-miR-150 as the miR-150 inhibitor in this study. First of all, we confirmed that subcutaneous injection of FAM-labeled LNA-anti-miR-150 could be absorbed by glomeruli and tubules (Fig. 2a). Two milligrams per kilogram of LNA-anti-miR-150 showed similar capability to inhibit the renal miR-150 expression effectively as 4 mg/kg of LNA-anti-miR-150 (Fig. 2b, c). The 2 mg/kg dosage was previously demonstrated as effective for the use of miR-192 inhibitor to treat DN mice by Putta et al. [16].

In terms of the effect of LNA-anti-miR-150 in SLE and LN mice, the titer of serological ds-DNA antibody, proteinuria, and severity of glomerular and tubulointerstial injury were all ameliorated compared to that of the mice given the scrambled LNA (Fig. 3). In regard to miR-150 function, miR-150 is demonstrated to control B cell development and function [32]. In addition, miR-150 also plays a key role in other immune cells. For example, miR-150 regulates the development of NK and iNKT cells [33]. miR-150 positively regulates differentiation and function in CD8+ T cells and negatively regulates the function of CD4+ T cells [34, 35]. However, we did not detect any change in CD19+ B cell, CD3+ total T cells, and subset CD4+ and CD8+ T cell in kidneys of LN mice. Therefore, the renal protective effect of LNA-anti-miR-150 in LN mice prompted us to investigate the underlying mechanisms. Based on our previous findings in vitro [9], we investigated the effect of LNA-anti-miR-150 on the expression of renal fibrosis-associated genes. We found that LNA-anti-miR-150 decreased renal overexpression of pro-fibrotic genes including *TGFβ1*, *α-SMA*, and *FN* on mRNA and protein levels (Fig. 4). These three pro-fibrotic genes are well known for promoting the progress of organ fibrosis [36]. We also found a reversal in reduction of anti-fibrotic gene *SOCS1* in both mRNA and protein levels (Fig. 5). The attenuation of renal fibrosis by miR-150 inhibition in LN mouse kidneys was supported by our previous data in vitro [9] and other groups’ findings in miR-150 deletion mice with renal fibrosis 8 weeks after ischemia and reperfusion in vivo [14].

In addition to the renal anti-fibrotic roles of LNA-anti-miR-150, we also found that LNA-anti-miR-150 decreased the production of cytokines (Fig. 6a) and the infiltration of macrophages as indicated by two biomarkers, F4/80 and CD68, on western blotting analysis and immunostaining (Fig. 6b, c). The role of macrophages has recently been reported in renal fibrosis. Renal macrophages may initiate inflammatory response to small immune complexes or albuminuria in the kidney [37, 38]. Activation of fibroblast by macrophages and direct macrophage-myofibroblast transition contribute to macrophage-rich acute inflammation and promote myofibroblast accumulation and renal fibrosis in IgA nephropathy and crescentic glomerular nephritis [39]. These findings supported our observations of the increased macrophage infiltration in LN mouse kidneys in this study. Our results suggest that renal protective mechanisms of LNA-anti-miR-150 against renal fibrosis in LN mice may also be mediated by modulating macrophages in kidneys.

**Fig. 8:**
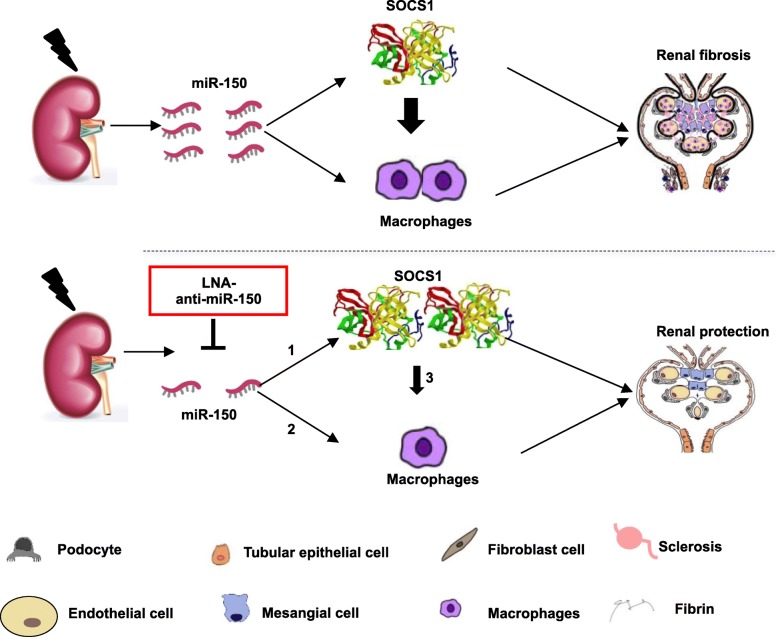
Schematic mechanism model of renal protective role of LNA-anti-miR-150. Renal miR-150 increased in lupus nephritis (LN). Renal protective roles of LNA-anti-miR-150 in LN might be mediated by three possible pathways: (1) direct anti-fibrotic role of SOCS1, (2) direct inhibition of macrophages infiltration, and (3) inhibition of renal macrophage filtration through increasing SOCS1

Aside from animal study of miR-150-modulated fibrotic genes and macrophages, we also explored the expression of these genes in human subjects. We found similar phenomena of miR-150, fibrosis-related proteins, and infiltrations of macrophages in renal biopsies from untreated new onset Chinese LN patients (Fig. 7). Our findings from LN mice and human subjects suggest that targeting nucleic acid might be a novel avenue to treat LN in addition to currently available steroids, immunosuppressants, and monoclonal antibodies. The corresponding mechanisms of the renal protective role of miR-150 inhibitor may be mediated by three pathways: (1) direct anti-fibrotic role of SOCS1, (2) direct inhibition of macrophage infiltration, and (3) inhibition of renal macrophage filtration through increasing SOCS1 (Fig. 8).

## Conclusions

LNA-anti-miR-150 ameliorated kidney injury of LN by decreasing the synthesis of pro-fibrotic proteins and increasing the production of anti-fibrotic SOCS1 protein. In addition, the renal protective role may also be associated with inhibiting the activation of kidney resident macrophages. Therefore, LNA-anti-miR-150 may serve as a promising novel agent for treating LN in the future.

## Supplementary information

**Additional file 1: Table S1.** The clinical characteristics of LN patients. **Table S2.** The clinical characteristics of normal control human subjects. **Table S3.** Antibodies used in western blotting and immunofluorescence staining. **Table S4.** Sequence of primers used in qPCR. **Table S5.** Sequence of LNA-anti-miR-150 and Scrambled LNA.

**Additional file 2: Figure S1.** Study design. **Figure S2.** The safety of LNA-anti-miR-150 as a therapeutic agent for LN. **Figure S3**. The effect of LNA-anti-miR-150 on the infiltration of B and T cells in kidneys of LN mice. **Figure S4**. Pathological features on renal biopsies of LN patients.

## Data Availability

The datasets used and/or analyzed during the current study are available from the corresponding author on reasonable request.

## Abbreviations

ACR: Albumin/ creatinine ratio
AKI: Acute kidney injury
ALT: Alanine aminotransferase
ANA: Anti-nuclear antibody
AST: Aspartate amino transferase
BUN: Blood urea nitrogen
C1q: Complement1q
DN: Diabetic nephropathy
ds-DNA: Anti-double strand DNA
ELISA: Enzyme-linked immunosorbent assay
ESRD: End stage renal disease
FN: Fibronectin
HPF: High power field
I/R: Ischemia/reperfusion
IL6: Interleukin-6
INF γ: Interferon-γ
LN: Lupus nephritis
LNA: Locked nucleic acid
LPS: Lipopolysaccharide
miR: MicroRNA
PAS: Periodic acid-Schiff
RT: Room temperature
SC: Subcutaneously
Scr: Serum creatinine
SLE: Systemic lupus erythematosus
SOCS1: Suppressor of cytokine signal 1
TGF-β1: Transforming growth factor-β1
TNFα: Tumor necrosis factor-α
WT: Wild type
α-SMA: α-Smooth muscle antibody
