# Supplementary material for: LNA-anti-miR-150 ameliorated kidney injury of lupus nephritis by inhibiting renal fibrosis and macrophage infiltration
Source: Arthritis Res Ther. 2019 Dec 11;21:276. doi: 10.1186/s13075-019-2044-2 (PMC6907329; doi:10.1186/s13075-019-2044-2)
Supplement: Supplementary file 1 — Additional file 1: Table S1. The clinical characteristics of LN patients. Table S2. The clinical characteristics of normal control human subjects. Table S3. Antibodies used in western blotting and immunofluorescence staining. Table S4. Sequence of primers used in qPCR. Table S5. Sequence of LNA-anti-miR-150 and Scrambled LNA. [file 13075_2019_2044_MOESM1_ESM.pdf]

Table S1. The clinical characteristics of LN patients

Pt. #	Age	Gender	SLEDAI (score)	eGFR (ml/min/1.73m^2)	Urine URTP (g/day)	Blood Alb (g/L)	ANA	ds-DNA	IgG (g/L)	Lymphs (10^6/uL)	CD4 (counts/uL)	C4 (g/L)	Renal biopsy AI (score)	CI (score)
1	52	F	22	44	5.8	24.3	2+	1+	7.3	1.65	3037	0.07	13	5
2	26	F	26	138	0.5	20.1	2+	1+	28.3	0.11	254	0.17	10	0
3	40	F	18	46	16.1	21.0	2+	-	9.3	0.80	541	0.15	13	2
4	40	F	20	120	0.2	19.5	2+	-	19.9	1.19	556	0.02	7	0
5	35	F	22	50	2.5	21.5	2+	1+	12.4	1.80	178	0.03	10	3
6	18	F	36	221	8.9	15.8	2+	-	12.6	1.38	317	0.03	1	0
7	49	F	21	112	3.5	24.4	2+	1+	12.6	0.75	283	0.03	4	1
8	36	F	30	123	2.5	28.3	2+	1+	21.7	0.87	95	0.05	4	0
9	44	F	14	94	2.6	28.3	1+	-	18.1	1.22	273	0.25	8	6
10	22	F	22	139	15.8	15.0	2+	2+	7.6	2.17	-	0.07	14	0

Pt, patient; SLEDAI, systemic lupus erythematous diseases activity index; eGFR, estimated glomerular filtration rate based on CKD-EPI formula; URTP, urinary total proteinuria per day; Alb, albumin; ANA, antinuclear antibodies; ds-DNA, double strands DNA; IgG, immunoglobulin G; Lymphs, lymphocyte counts; C4, complement 4; AI, activity index; CI, chronicity index.

Table S2. The clinical characteristics of normal controls

Normal control	Age	Gender	eGFR (ml/min/1.73m^2)	Urine		Serum
				Proteinuria	Gravity	Alb (g/L)
1	32	F	106	±	1.024	43.6
2	50	F	139	-	1.016	37
3	47	F	86	-	1.029	40.9
4	53	F	122	1+	1.016	42.3
5	53	F	120	-	1.01	41.7
6	47	F	117	±	1.025	45.2
7	59	F	115	-	1.009	36.4
8	46	F	104	-	1.016	43
9	54	F	122	-	1.021	40.7
10	52	F	123	±	1.024	40

eGFR, estimated glomerular filtration rate based on CKD-EPI formula; Alb, albumin.

Table S3. Antibodies used in western blotting and immunostaining

Protein	Company	Catalog	Host	Application	Dilution
C1q	Abcam	ab89901	Rabbit	IF	1:300
α-SMA	Sigma	19245	Mouse	WB	1:1000
				IF/IHC	1:200
TGF-β1	novusbio	NBP1-80289	Rabbit	WB	1:1000
				IF/IHC	1:200
Fibronectin	Abcam	ab199056	Rabbit	WB	1:1000
				IF/IHC	1:1000
SOCS1	Bioss	BS-0113R	Rabbit	WB	1:1000
				IF/IHC	1:200
CD68	Santa Cruz Biotechnology	SC-20060	Mouse	IF/IHC	1:200
F4/80	Santa Cruz Biotechnology	SC-25830	Rabbit	WB	1:1000
				IHC	1:200
CD19	Servicebio	GB11061-1	Rabbit	IF	1:400
CD3	Servicebio	GB11014	Rabbit	IF	1:200
CD4	Servicebio	GB11064	Rabbit	IF	1:800
CD8	Servicebio	GB11068	Rabbit	IF	1:200
GADPH	Santa Cruz Biotechnology	SC-25778	Rabbit	WB	1:1000
α-TUBULIN	Santa Cruz Biotechnology	SC-5286	Mouse	WB	1:1000
Goat anti Mouse IgG	Santa Cruz Biotechnology	SC-2039	Goat	WB	1:8000
Goat anti Rabbit IgG	Thermo Fisher Scientific	31460	Goat	WB	1:10000
Alexa Fluor® 488	Thermo Fisher Scientific	A11029	Goat	IF	1:200
Alexa Fluor® 568	Thermo Fisher Scientific	A11036	Goat	IF	1:200

a-SMA, a-smooth muscle actin; TGF-β, transforming growth factor β1; SOCS1, suppressor of cytokine signaling 1; CD, cluster of differentiation; GADPH, glyceraldehyde-3-phosphate dehydrogenase; WB, western blotting; IF, immunofluorescence staining; IHC, immunohistochemical staining.

Table S4. Sequence of primers used in qPCR

Gene	Forward(5'-3')	Reverse(5'-3')
αSma	TCAGGGAGTAATGGTTGGAATG	GGTGATGATGCCGTGTT
Tgfβ1	CCCGAAGCGGACTACTATGC	CATAGATGGCGTTGTTGCGG
Fibronectin	ATGTGGACCCCTCCTGATAGT	GCCCAGTGATTTCAGCAAAGG
Socs1	CTGCGGCTTCTATTGGGGAC	AAAAGGCAGTCGAAGGTCTCG
Il6	CTGCAAGAGACTTCCATCCAGTT	AGGGAAGGCCGTGGTTGT
Ifnγ	CCAGCGCAAAGCAATAAGTG	GGCCTCGAAACGGATTCTG
Tnfα	CAGCCGATGGGTTGTACCTT	GGCAGCCTTGTCCCTTGA
Actin	TTCCTTCTTGGGTATGGAAT	GAGCAATGATCTTGATCTTC
miR-150	TCTCCCAACCCTTGTACCAGTG	
Sno202	GCTGTACTGACTTGATGAAAGTACT (mmu)	
U6	GCTTCGGCAGCACATATACTAAAAT(hsa)	

aSma, a-smooth muscle actin; Tfgβ1, transforming growth factor β1; Sosc1, suppressor of cytokine signaling 1; Inf γ, interferon γ; Il6, interleukin ; Tnfα, tumor necrosis factor α

Table S5. Sequence of LNA-anti-miR-150 and Scrambled LNA

Name	5'-3'
LNA-anti-miR-150	TACAAGGGTTGGGAG
Scrambled LNA	TAGAAGGGTGGTGAC

LNA, locked nucleic acid
