# Supplementary material for: LNA-anti-miR-150 ameliorated kidney injury of lupus nephritis by inhibiting renal fibrosis and macrophage infiltration
Source: Arthritis Res Ther. 2019 Dec 11;21:276. doi: 10.1186/s13075-019-2044-2 (PMC6907329; doi:10.1186/s13075-019-2044-2)
Supplement: Supplementary file 2 — Additional file 2: Figure S1. Study design. Figure S2. The safety of LNA-anti-miR-150 as a therapeutic agent for LN. Figure S3. The effect of LNA-anti-miR-150 on the infiltration of B and T cells in kidneys of LN mice. Figure S4. Pathological features on renal biopsies of LN patients. [file 13075_2019_2044_MOESM2_ESM.pdf]

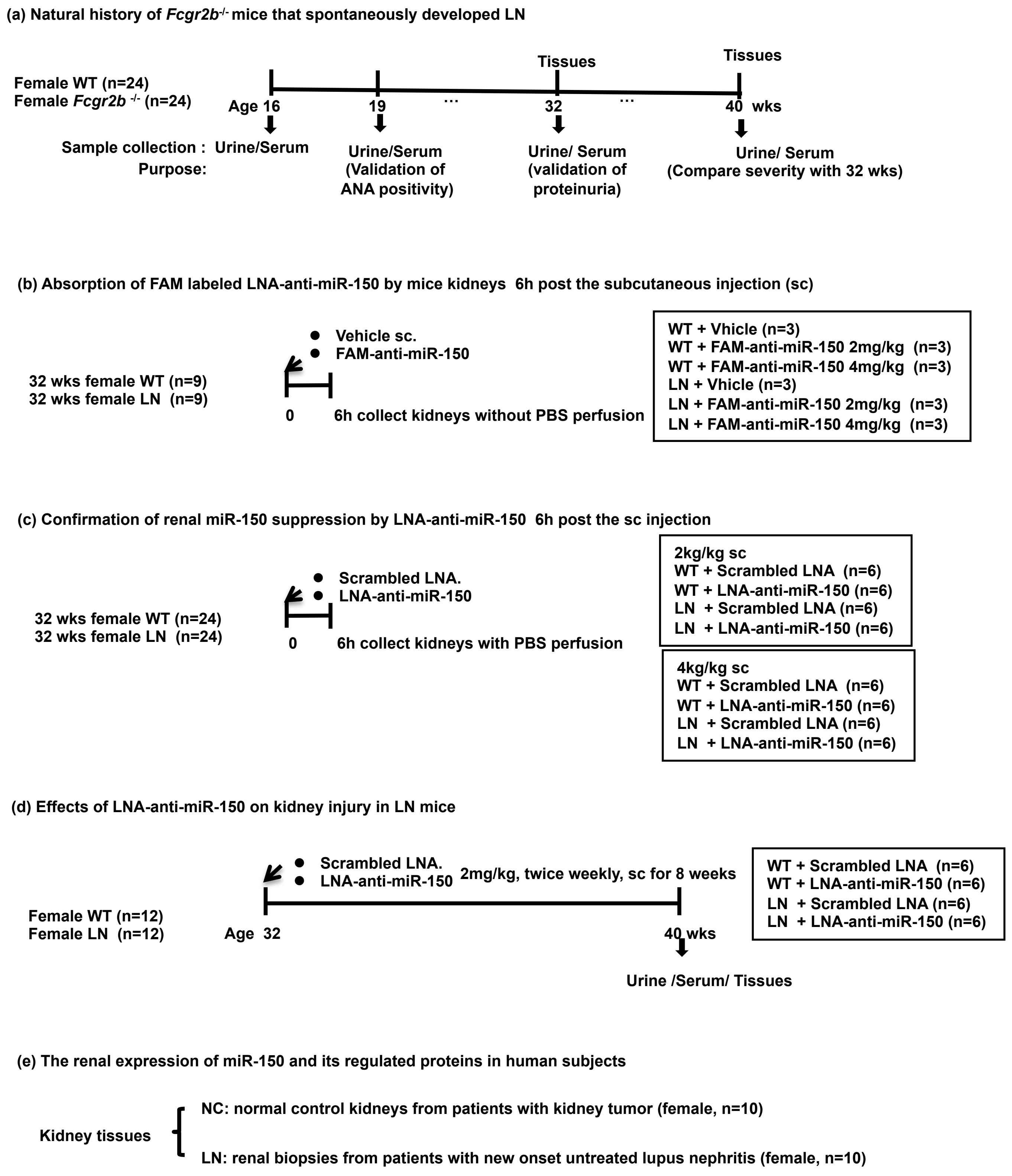

Figure S1. Study design. (a) Natural history of Fcgr2b−/−spontaneous lupus nephritis (LN). (b) The absorption of FAM-labeled locked nucleic acid (LNA)-anti-miR-150 by kidney 6 hours after the subcutaneous injection. (c) The suppression of renal miR-150 levels (d) The effect of LNA-anti-miR-150 on kidney injury in LN mice. (e) Renal miR-150 expression in LN patients.

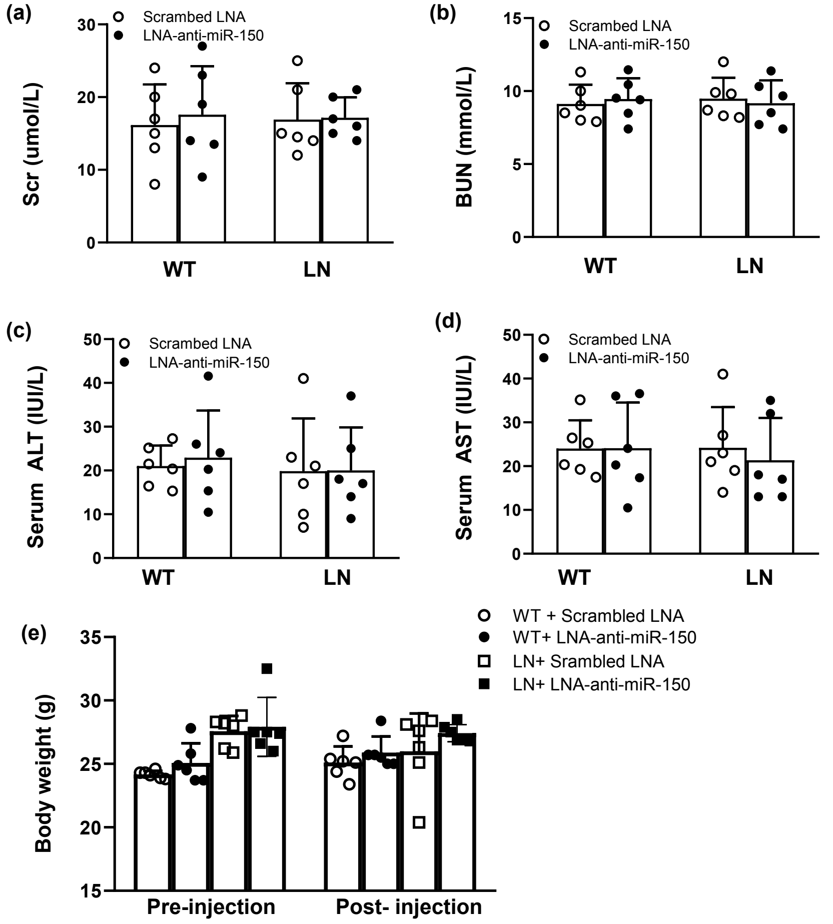

Figure S2. The safety of LNA-anti-miR-150 as a therapeutic agent in LN mice. (a) Serum creatinine (Scr) , (b) blood urea nitrogen (BUN), (c-d) hepatic enzymes was assayed by an Architect c1600 device and (e) body weight was measured in WT and LN mice treated with the scrambled LNA or LNA-anti-miR-150 (subcutaneous injection twice weekly for eight weeks, six mice per group). Data are expressed as mean ± SD from six mice per group. Statistical significance was determined using two-way ANOVA (#$p<0.05$, LN vs. WT. *$p<0.05$, LNA-anti-miR-150 vs. the scrambled LNA).

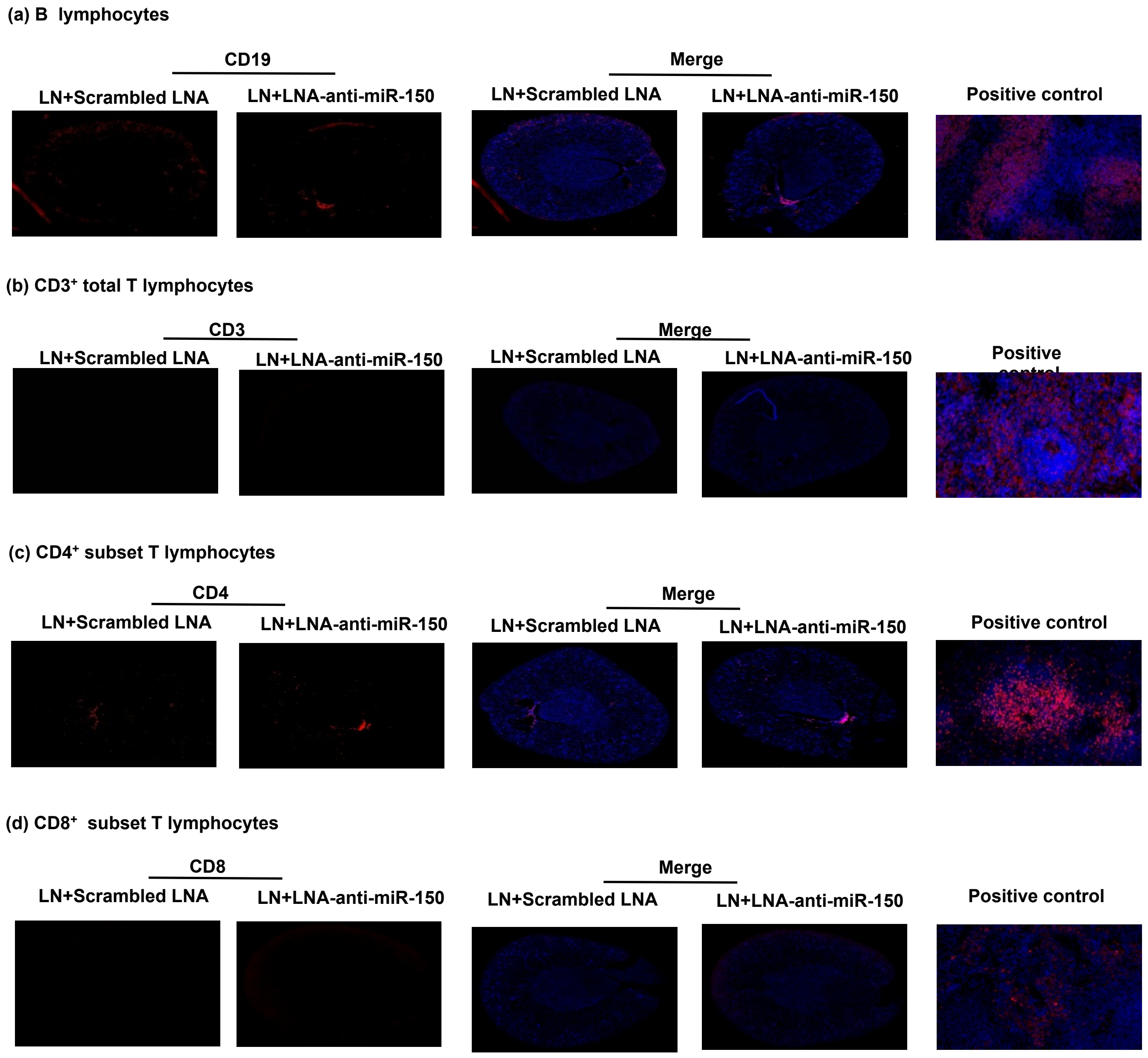

Figure S3. The effect of LNA-anti-miR-150 on the infiltration of B and T cells in kidneys of LN mice. Renal expression of B lymphocytes (a) and $CD3^+$ total T lymphocytes (b) as well as subset $CD4^+$ T (c) and $CD8^+$ T lymphocytes (d) was detected by immunoflurescent (IF) staining in LN mice treated with the scrambled LNA or LNA-anti-miR-150 (subcutaneous injection twice weekly for eight weeks, six mice per group) (Magnification, 1.5X). Spleen was used as positive control of IF staining for B and T lymphocytes (magnification, 200X).

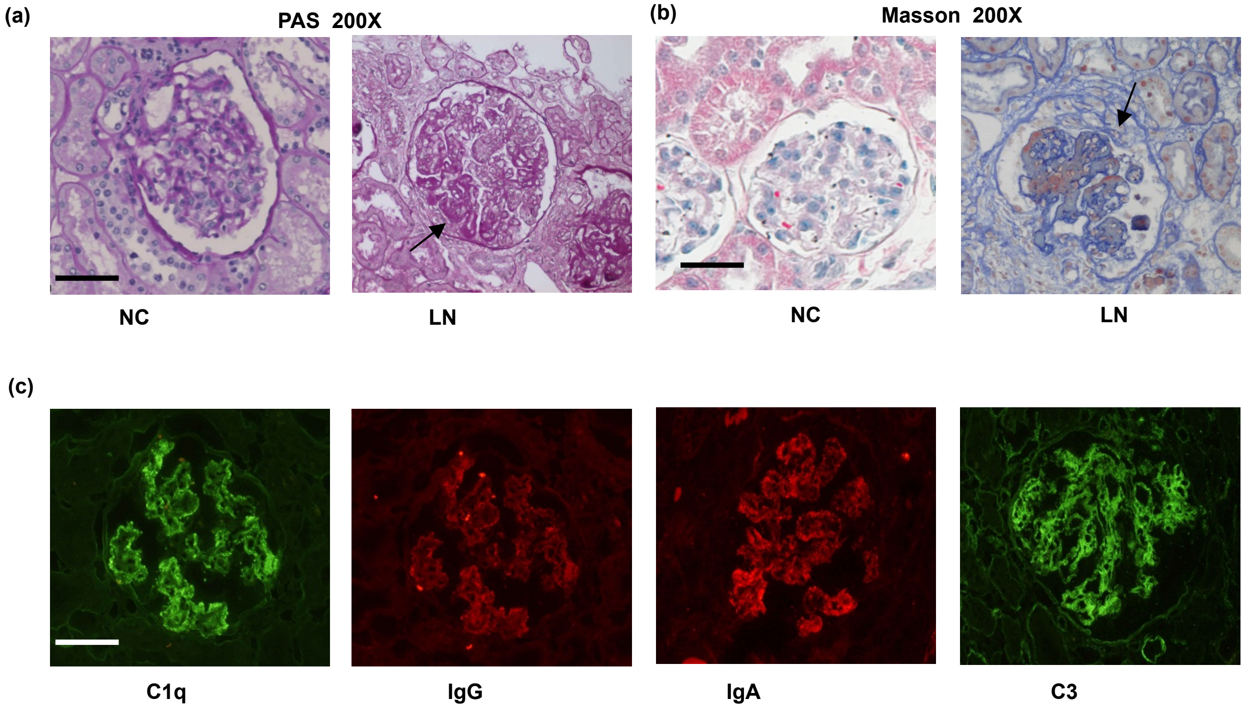

Figure S4. Pathological features on renal biopsies of LN patients. (a-b) PAS and Masson staining of paraffin-embedded kidney sections taken from renal biopsies of LN patients showed typical endocapillary proliferation, lobular capillary tufts, and small crescents (black arrows) compared to normal control kidney tissue (NC). (c) Immunofluorescent staining of renal biopsies from LN patients showed “Full house” with positive stained C1q, IgG, IgA, and C3 (magnification × 200, bar=60μm).
